# Melanin: the biophysiology of oral melanocytes and physiological oral pigmentation

**DOI:** 10.1186/1746-160X-10-8

**Published:** 2014-03-24

**Authors:** Liviu Feller, Aubrey Masilana, Razia AG Khammissa, Mario Altini, Yusuf Jadwat, Johan Lemmer

**Affiliations:** 1 Department of Periodontology and Oral Medicine, University of Limpopo (Medunsa Campus), Pretoria, South Africa; 2 Division of Anatomical Pathology, School of Pathology, University of the Witwatersrand, Johannesburg, South Africa; 3 Department Periodontology and Oral Medicine, Box D26 School of Dentistry, Medunsa 0204, South Africa

**Keywords:** Melanin, Oral melanocytes, Pigmentation, Melanocyte stem cell, Keratinocyte, Melanosome, Pigment transfer

## Abstract

The presence of melanocytes in the oral epithelium is a well-established fact, but their physiological functions are not well defined. Melanin provides protection from environmental stressors such as ultraviolet radiation and reactive oxygen species; and melanocytes function as stress-sensors having the capacity both to react to and to produce a variety of microenvironmental cytokines and growth factors, modulating immune, inflammatory and antibacterial responses. Melanocytes also act as neuroendocrine cells producing local neurotransmitters including acetylcholine, catecholamines and opioids, and hormones of the melanocortin system such as proopiomelanocortin, adrenocorticotropic hormone and α-melanocyte stimulating hormone, that participate in intracellular and in intercellular signalling pathways, thus contributing to tissue homeostasis.

There is a wide range of normal variation in melanin pigmentation of the oral mucosa. In general, darker skinned persons more frequently have oral melanin pigmentation than light-skinned persons. Variations in oral physiological pigmentation are genetically determined unless associated with some underlying disease.

In this article, we discuss some aspects of the biophysiology of oral melanocytes, of the functions of melanin, and of physiological oral pigmentation.

## Introduction

While under physiological conditions the number of melanocytes in the oral epithelium is the same regardless of racial/ethnic origin [1], the colour of oral mucosa varies between persons and is determined by several factors including the number and melanogenic activity of the melanocytes in the basal cell layer of the epithelium, differences in number, size, and distribution of melanosomes, differences in the type of melanins, and the masking effect of heavily keratinized epithelium [2-4]. The variations in melanin colourisation of the oral mucosa will be determined against a background of the degree of vascularization of the tissues and by the level of haemoglobin in the blood [2].

Melanocytes containing melanin are present in the basal cell layer of the epithelium even at those oral mucosal sites that show no visible signs of melanin pigmentation [5-7]. Oral melanocytes may or may not produce melanin, but as in the case of the skin, the amount of melanin they produce is genetically determined [2,6]. There are substantial variations in the degree of melanin pigmentation between persons of different racial/ethnic groups and between persons of the same racial/ethnic group, and these variations are normal [2,8,9]. Physiological/racial melanin pigmentation of the oral mucosa is common in black persons [7], and is more frequent in darker skinned whites (Caucasians) than in lighter skinned whites [10]. The oral mucosal melanin pigmentation may be patchy or uniform and most commonly affects the gingiva [9].

Melanocytes produce melanin in membrane-bound organelles termed melanosomes. Melanosomes have all the proteins and enzymes necessary for melanin biosynthesis, for maintaining the structure of the melanosome, and for the maturation of the immature pre-melanosome into a mature melanosome producing melanin [11,12]. As the melanosomes mature intracellularly, they are transported via microtubuli to the surfaces of the elongated dendrites of the melanocytes whence they are ultimately transferred to the keratinocytes in the ‘keratinocyte melanin unit’ [11,12]. Within the keratinocytes, the melanin is preferentially localised within the nuclei forming protective barriers known as supranuclear ‘caps’ oriented in such a way that they shield the nuclear DNA from ultraviolet (UV) radiation [13].

Non-physiological alterations in melanin pigmentation of the oral mucosa are related to genetic, metabolic, endocrine, chemical or physical factors, to infective agents and to inflammatory or neoplastic processes [6,9]. It has been reported that in about 30% of cases, oral mucosal melanoma develops at sites of hyperpigmentation [2,14]. However, the nature of the hyperpigmented pre-melanoma is obscure.

Most of our knowledge about the regulation and biology of oral melanocytes is derived from data obtained from research on epidermal melanocytes which are histologically and ultra-structurally similar [15,16]. However, when extrapolating data from epidermal melanocytes, one needs to bear in mind that as a rule, oral mucosal melanocytes under physiological circumstances are said to be less metabolically active [6]. Nevertheless, in some cases oral melanocytes may be inherently metabolically active, and in other cases an increase in metabolic activity may be in response to environmental triggers such as hormones, inflammation or injury [6].

The aim of this short review is to discuss some aspects of the biology and physiology of oral melanocytes, the function of melanin and critically to evaluate the concept of intraoral physiological pigmentation.

### The origin of oral melanocytes

Melanocytes are melanin-producing cells originating from the neural crest [17]. During development melanocyte stem cells migrate from the neural crest to the skin and to mucous membranes. Active melanocytes are present in the stria vascularis of the cochlea, in the leptomeninges, in the substantia nigra and locus coerulus of the brain, and in the heart, where they play a number of as yet ill-defined roles [13,18].

Melanocyte stem cells have the capacity for self-renewal and for differentiation and thus can maintain the population of mature melanocytes. In the epidermis, the melanocyte stem cells reside in the bulge region of hair follicles [19,20], but the niche in which they reside in the oral mucosa is unknown.

Epidermal melanocyte stem cells give rise to transient-amplifying melanocyte precursors that settle without the stem cell niche where they differentiate into mature melanin producing melanocytes [19,21-24]. Stem cell factor (SCF) and its tyrosine kinase receptor C-kit signalling pathways are critical for epidermal melanocyte development during embryogenesis [17,20], notch signalling pathways are essential in the maintenance of adult melanocyte stem cells, and thus for melanocyte homeostasis [20], endothelin 1 plays a role in the differentiation of melanocyte precursors, and micropthalmia-associated transcription factor (MITF) with its cAMP response element play a critical role in melanogenesis [25].

In the skin, migration of precursor melanocytes from the dermis to their final destination in the basal cell layer of the epithelium is mediated by c-Kit/SCF, endothelin 1 and 3, hepatocyte growth factor (HGF) and basic fibroblast growth factor (bFGF). These precursor dermal melanocytes, as they pass through the basement membrane, express E-cadherin which later facilitates intercellular communication with neighbouring keratinocytes in the basal cell layer of the epithelium [1]. Sometimes, melanocyte precursors on their way to the epithelium may become arrested in the lamina propria/dermis, and as they have the capacity to produce melanin, if they are aggregated they will form nevi [26]. The functional activity of melanocytes, both those in the basal cell layer of the epithelium and those that may have become arrested in the lamina propria is influenced by signals from the neighbouring fibroblasts [13,27,28].

### The keratinocyte-melanocyte unit

Mature melanocytes are elongated dendritic cells residing in the basal cell layer of the epithelium. They contain all the proteins required for melanin biosynthesis and for the structural maturation of melanosomes [12], including tryosinase (TYR), tyrosinase-related proteins-1 (TRP-1) and TRP-2, gp 100, and melanoma antigen recognizable by T lymphocytes (MART-1) [13,29-31].

In the basal cell layer of the epithelium the ratio of melanocytes to keratinocytes ranges from 1:10 [30] to 1:15 [6]. Melanocytes and keratinocytes form epidermal melanin units, each unit consists of one melanocyte and a group of about 36 neighbouring keratinocytes. The melanosomes produced by melanocytes are disseminated via a network of melanocytic dendritic processes to the keratinocytes of the epidermal melanin unit [25,32]. It appears that keratinocytes have some control over the process of dendritic melanosome transfer since the capacity of keratinocytes to phagocytose the melanosomes is influenced by the degree of activation of the protease-activated receptor 2 (PAR-2) on the surface of keratinocytes [33].

It has been suggested that through biological mediators present in the melanosomes transferred to keratinocytes, melanocytes can influence the functional activities of keratinocytes [13], and keratinocytes *via* an array of paracrine-like biological mediators that they secrete, have the capacity to regulate melanocyte melanogenesis [13,25,34]. These mediators include α-melanocyte stimulating hormone (α-MSH), adrenocorticotropin hormone (ACTH), β-endorphin, bFGF, endothelins, HGF and SCF [35].

However, such a mechanistic description is an oversimplification as the ratio of keratinocytes to melanocytes is different at different stages of growth and development, and in adulthood the ratio is variable being determined by biological mediators secreted in the local microenvironment [33]. Furthermore, the numerical density of melanocytes in the epithelium varies in different parts of the skin or oral mucosa, and between the same skin or mucosa sites in different persons, regardless of their racial/ethnic origin [36]. It appears that Langerhans cells in the epithelium, and fibroblasts in the subepithelial connective tissue play an important role in maintaining the functional activity of the epidermal melanin unit [30,31,33].

The adherence of melanocytes to keratinocytes is by means of tight junctions where they co-express E-cadherin cell adhesion molecules and by gap junctions [33]. E-cadherin supresses melanocyte proliferation, but a switch from E-cadherin to N-cadherin owing to metabolically or traumatically triggered events in the microenvironment obviates the melanocytes from growth suppression. Melanocytes expressing N-cadherin can freely proliferate, migrate and self-aggregate in nests, and exhibit longevity [32].

### The function of oral melanocytes

The functions of melanocytes are not fully understood, but it is clear that the melanin that they produce determines the colour of skin, hair and eyes [37], and provides protection from stressors such as UV radiation, reactive oxygen species (ROS) and free radicals in the environment. Melanins also have the capacity to sequester metal ions and to bind certain drugs and organic molecules [32,38].

The colour of skin and probably of any pigmented part of the oral mucosa is genetically determined by the number and size of the melanosomes and the type of melanin (eumelanin, pheomelanin) that they produce. Environmental factors have only a modifying influence on skin colour, though on an evolutionary scale this influence can have a more profound effect. Melanosomes vary in size and contain both eumelanin and pheomelanin. More, larger eumelanin impart a dark colour to the skin [33]. Small, few pheomelanin-containing melanosomes are associated with light skin, and the spectrum of size and number of melanosomes and the balance of eumelanin to pheomelanin within the melanosomes, will determine all the other colour variations [32].

As melanin synthesis is an oxygen dependent process, paradoxically, it also generates ROS that may accumulate in the melanocytes and cause DNA damage, and in fact UV radiation exaggerates the production of ROS during the biosynthesis of melanin and more particularly pheomelanin [39]. Thus, melanin possesses both antioxidant and ROS-dependent cytotoxic properties [18].

Quinones and semiquinones which are intermediates of melanogenesis are toxic or mutagenic with the potential to cause cytogenetic instability [40]; and L-dopa, another intermediate of melanogenesis, has the capacity to inhibit the production of proinflammatory cytokines by T lymphocytes and monocytes, thus downregulating immune and inflammatory responses [38,40].

Melanins produced by melanocytes residing in the basal cell layer of the gingival epithelium have the capacity to neutralize ROS generated by dentogingival plaque-induced inflammation in the periodontal microenvironment [16]. Interestingly, a recent report shows that markers of gingival inflammation are reduced in subjects with pigmented gingiva compared to subjects with non-pigmented gingiva, despite comparable dentogingival plaque levels in both groups of subjects. However, one needs to bear in mind that the sulcular and junctional epithelium, in contrast to the keratinized epithelium usually does not harbour any melanocytes [16].

Melanosomes contain lysosomal enzymes including α-mannosidase, acid phosphatase, β-N acetylglycosaminidase, β-galactosidase, and acid lipase that can degrade bacteria [41]. Melanin itself can neutralise bacteria-derived enzymes and toxins, and since it has strong binding properties, it can also act as a physical barrier against microorganisms [41]. Furthermore, melanocytes can act as antigen presenting cells, can stimulate T-cell proliferation, and can phagocytose microorganisms [32,41]. Thus, melanocytes and their products have the capacity to inhibit proliferation of bacterial and fungal microorganisms [41].

As keratinocytes ascend through the cell layers of the epithelium and are shed, their melanosomal membranes undergo degradation with release of melanin ‘dust’ which becomes entangled in the keratin filaments of the desquamating surface cells. This melanin dust inactivates pathogenic chemicals, microbial toxins and other biologically active molecules [18]. Therefore, melanocytes and melanins may be viewed as an integral part of the innate immune system with a role in neutralising the products of superficial bacterial and fungal infective agents [41].

It is possible that from an evolutionary point of view, the primary role of melanocytes is not to produce melanin, for melanin does not confer a selective advantage to the organisms, but that melanin production is only a secondary specialization so melanocytes must have other more important functions to perform [13]. Melanocytes in skin, and perhaps in oral mucosa express genes encoding corticotropin releasing factor (CRF), proopiomelanocortin (POMC), ACTH, β endorphins, α-MSH and melanocortin-1 receptor (MC1R). These elements of the skin melanocortin system have the capacity to neutralize external noxious agents, to mediate local antimicrobial and immune responses, and to mediate local nociception [34,42]. UV radiation induces the generation of CRF, POMC and α-MSH by cutaneous keratinocytes and melanocytes, bringing about an increase in melanin biosynthesis [43], imparting protection against solar radiation [13].

Melanocyte-derived α-MSH, ACTH, and other peptides of POMC, stimulate the MC1R of neighbouring melanocytes, activating an intracellular signalling cascade involving the second messenger cAMP and the MITF [34]. The α-MSH/MC1R/cAMP/MITF pathway controls the transcription of tyrosine and is therefore essential for melanogenesis [25], and also determines the type and quantity of the melanin produced [1,13].

### Regulation of melanogenesis

Melanogenesis comprises a process of oxidation of the amino acid tyrosine and derived aromatic compounds, forming two main types of polymeric phenolic compounds. Large, irregular granules of eumelanin which are black-brown in colour and smaller, more regular granules of pheomelanin which are yellow-red in colour [41]. Melanocytes are capable of producing both eumelanin and pheomelanin, and the proportion of the two melanins produced by a particular melanocyte is a function of availability of tyrosine, of reducing agents, and of the types of pigment enzymes expressed [1].

The melanocortin system through the cAMP/MITF pathway can stimulate proliferation of undifferentiated melanocytes, their maturation with fully formed dendritic processes, and upregulation of melanogenesis, thus increasing the formation of melanin [42,44]. α-MSH has the capacity to suppress inflammatory responses, because it can inhibit nuclear factor-κB (NF-κB) that regulates the expression of genes of proinflammatory cytokines. Thus, the same biological mediator of the melanocortin system that stimulates melanogenesis, also downregulates inflammatory responses [33].

It appears that the epidermal adrenergic signalling pathway plays a role in the regulation of skin pigmentation. Epidermal melanocytes express α1- and β2-adrenoceptors, the activation of which leads to an increase in melanin biosynthesis and a concurrent increase in the number and complexity of melanocytic dendrites. The adrenalin/β2-adrenoceptor/cAMP/MITF pathway like the α-MSH /MC1R/cAMP/MITF pathway has therefore the capacity to mediate melanin production and its dendritic distribution [44].

β-endorphin, an opioid peptide cleaved from POMC is positively associated with increased skin pigmentation. The β-endorphin/μ-opioid receptor/PKCβ isoform signalling pathway is expressed and functionally active, mediating differentiation and maturation of melanocytes with increased melanogenesis and dendricity [36,45]. To the best of our knowledge, the role of melanocortin, adrenergic and opioid systems in relation to melanogenesis in the oral epithelium is unknown.

Mediators of inflammation such as histamine and arachidonic acid metabolites trigger melanogenesis [41], and inflammatory cytokines such as TNF-α and IL-1α induce the secretion of melanogenic agents (SCF, HGF, bFGF, endothelins) by keratinocytes. Together these agents account for the melanin pigmentation sometimes observed in association with inflammatory conditions of skin or oral mucosa [41,46], such as oral lichen planus [6] and healing after periodontal surgery [47].

### Physiological oral pigmentation

Variation in oral physiological melanin pigmentation is ascribable to variations in the activity of melanocytes in the basal cell layer of the oral epithelium. Such oral pigmentation is more common in darker-skinned persons regardless of their racial/ethnic origin [10,16]. This strongly suggests that physiological oral pigmentation is determined by genetic factors associated with melanogenesis [15]. Microscopical examination of physiologically pigmented oral mucosae shows increased melanin in the basal cell layer and sometimes in the upper portion of the lamina propria within melanophages, or simply as extracellular melanin particles [15]. These microscopical features are very similar to those found in melanotic maculae and smoker’s melanosis [3].

Physiological oral pigmentation and pathological oral pigmentations that may be similar in appearance should be differentiated. Diseases that may be confused with physiological oral pigmentation include Addison’s disease, neurofibromatosis, oral melanotic maculae, oral mucosal melanoma, drug-induced oral mucosal pigmentation, and to a much lesser extent Kaposi sarcoma, vascular malformations and haemangioma of the oral mucosa [48].

**Figure 1:**
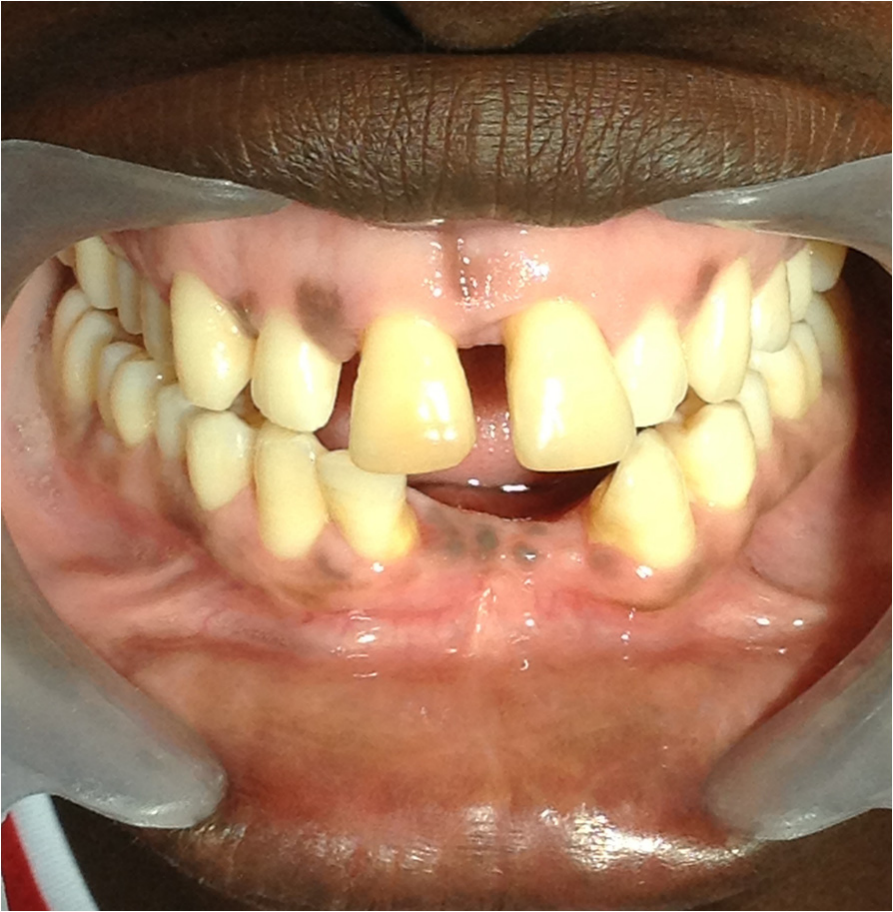
Multiple, light brown maculae with well-defined borders on the gingiva.

**Figure 2:**
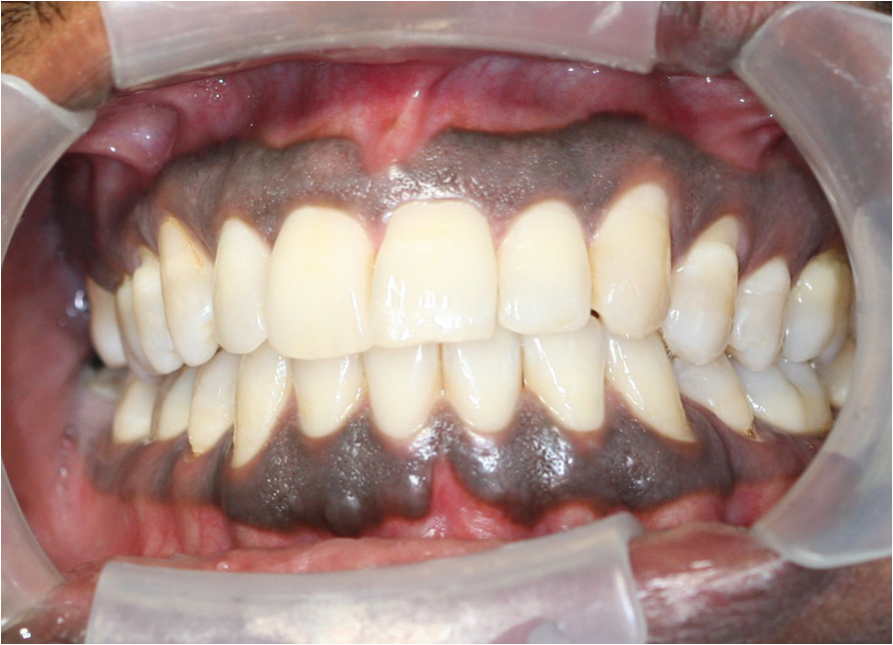
Physiological oral pigmentation on the gingiva presenting as bilateral, symmetrical, dark brown discolouration of the labial gingiva, including the marginal and papillary gingiva but not transgressing the mucogingival junction.

**Figure 3:**
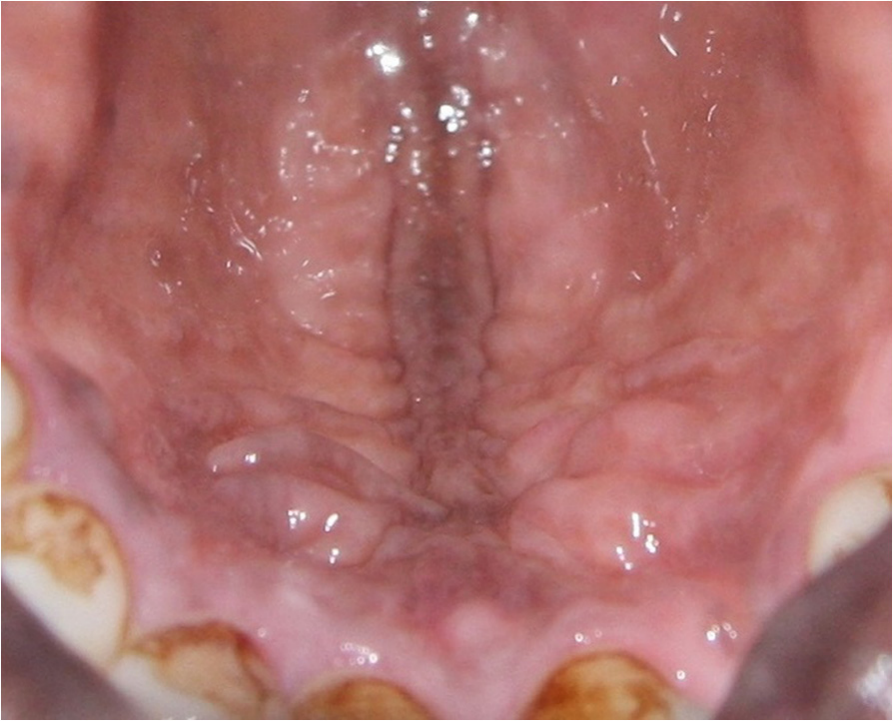
Diffuse, light brown physiological oral pigmentation on the hard palate.

**Figure 4:**
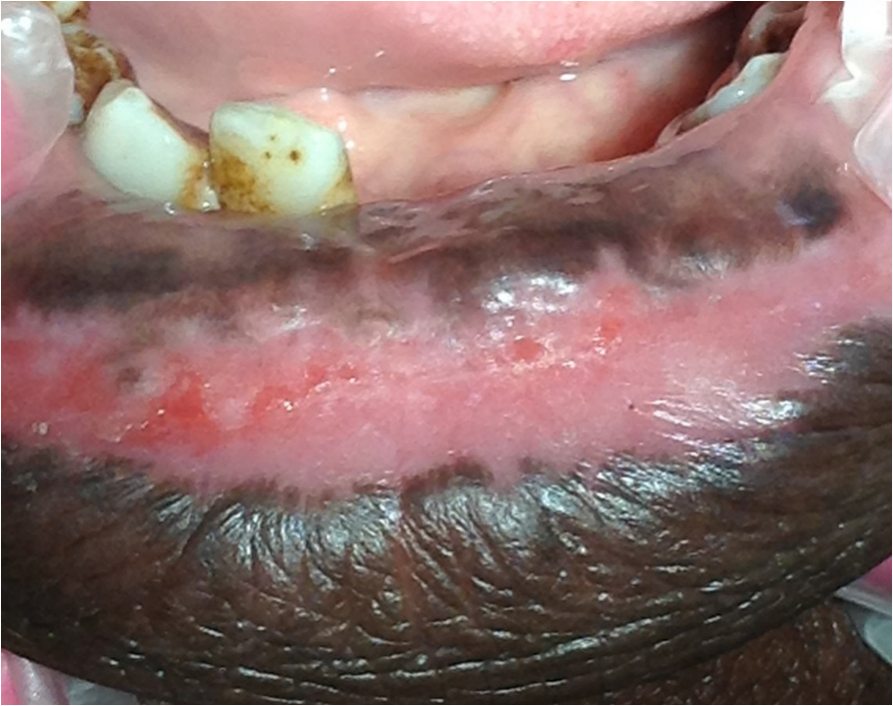
Uniform dark brown-black pigmentation of the lower lip and labial mucosa.

**Figure 5:**
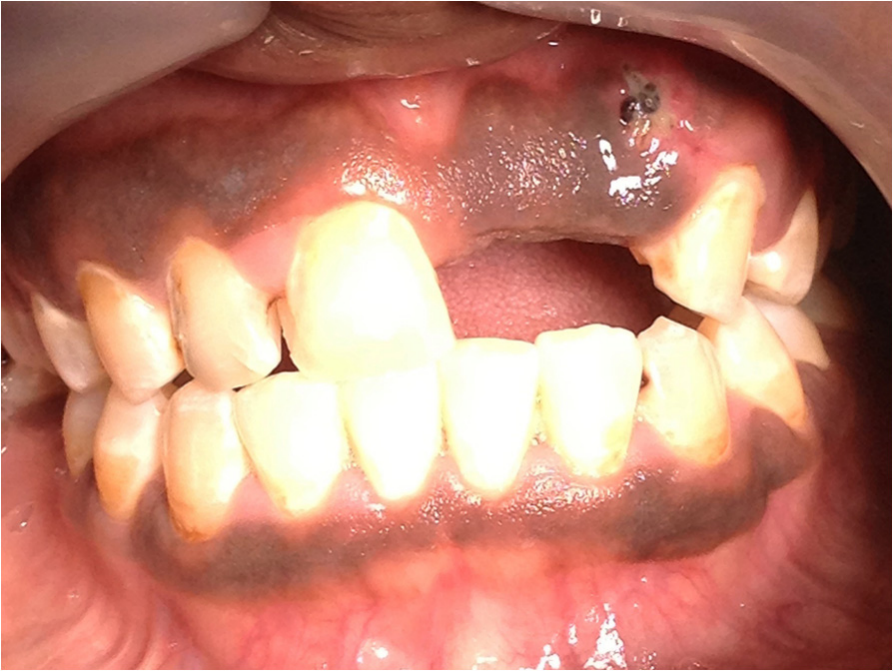
**Physiological oral pigmentation in a black male: a well demarcated brown band on the attached gingiva, not transgressing the mucogingival junction and not affecting the marginal/interdental papillary gingiva.** (The foreign body at the upper left is a post-biopsy suture).

**Figure 6:**
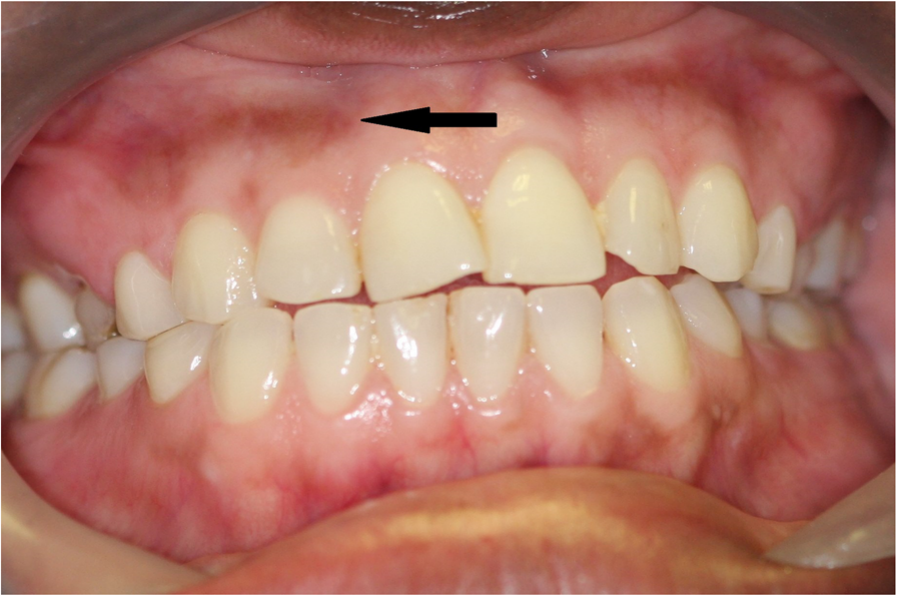
Light brown pigmentation of the alveolar mucosa not transgressing the mucogingival junction.

Physiological melanin pigmentation of the oral mucosa affects males and females equally, as asymptomatic, solitary or multiple brown maculae with well-defined borders (Figure 1) [49]. It may involve any part of the oral mucosa (Figures 2, 3, 4), but most frequently the gingiva [3,48,50]. In the gingivae, pigmentation when it occurs, is most commonly bilaterally symmetrical and does not transgress the mucogingival junction (Figure 2) [3,8,9,48,51], and in such cases sometimes the free gingiva is not pigmented (Figure 5) [3,7,48]. Occasionally, the alveolar mucosa is affected, and again the mucogingival junction is not transgressed (Figure 6). Oral pigmentation gradually appears during the first two decades of life [48] but affected subjects may be unaware of it [48,50].

The extent and intensity of physiological oral pigmentation increases with age [52,53], concurrently with an increase in the number of oral melanocytes [15], perhaps because of the effects of potentially melanogenic stimuli such as recurrent minor functional injury, inflammatory conditions, medication, or tobacco smoke are cumulative [3].

The global frequency of physiological oral pigmentation in different racial/ethnic groups is unknown; but it has been reported that about 95% of healthy black persons in the United States are affected; that pigmentation is more extensive in the anterior than in the posterior portion of the mouth; and that when the gingiva is affected the buccal/labial surfaces are more intensely pigmented than the lingual/palatal surfaces [7]. It has been reported in one study from South Africa that oral pigmentation occurs in 98% of black persons [54].

Eighty to a hundred percent of Australian Aborigines have physiological oral pigmentation with equal gender distribution [52]. By contrast Fry and Almeyda [55] have reported that only about 5% of white people in England have physiological buccal mucosal pigmentation. In general, it appears that persons with more darkly pigmented skin are more likely to have physiological oral pigmentation [7].

### Summary

Melanocytes can adjust and respond to biological, physical and chemical stimuli in their microenvironment, and can in turn generate physical and biochemical signals which may affect that microenvironment. Melanocytes have the capacity to mediate antimicrobial and immune responses and to act as neuroendocrine cells, and they produce melanin that provides protection from environmental stressors such as UV radiation, reactive oxygen species and free radicals. Why certain persons are more affected by physiological oral pigmentation is unknown, but certainly it occurs predominantly in darker skinned persons.

## Consent

Written informed consent was obtained for the publication of this report and any accompanying images.

## Competing interests

The authors declare that they have no competing interests.

## Authors’ contributions

Concept of paper was devised by LF. LF, AM, RAGK, MA, YJ, and JL wrote the manuscript. The manuscript was critically revised by LF, RAGK and JL. All authors read and approved the manuscript.
